# A case of ABO-incompatible blood transfusion treated by plasma exchange therapy and continuous hemodiafiltration

**DOI:** 10.1007/s13730-018-0307-4

**Published:** 2018-01-31

**Authors:** Akio Namikawa, Yuko Shibuya, Haruki Ouchi, Hiroko Takahashi, Yoshitaka Furuto

**Affiliations:** Department of Hypertension and Nephrology, NTT Medical Center Tokyo, 5-9-22 Higash-Gotanda, Shinagawa-ku, Tokyo, 141-8625 Japan

**Keywords:** Incompatible blood transfusion, Acute hemolytic reaction, Disseminated intravascular coagulation, Acute kidney injury, Plasma exchange therapy, Continuous hemodiafiltration

## Abstract

ABO-incompatible blood transfusion is potentially a life-threatening event. A 74-year-old type O Rh-positive male was accidentally transfused with 280 mL type B Rh-positive red blood cells during open right hemicolectomy, causing ABO-incompatible blood transfusion. Immediately after the transfusion, the patient experienced a hypotension episode followed by acute hemolytic reaction, disseminated intravascular coagulation and acute kidney injury. Plasma exchange therapy was performed to remove anti-B antibody and free hemoglobin because they caused acute hemolytic reaction, disseminated intravascular coagulation, and acute kidney injury. Free hemoglobin levels decreased from 13 to 2 mg/dL for 2 h. Continuous hemodiafiltration was used to stabilize hemodynamics. The patient was successfully treated for acute hemolytic reaction, disseminated intravascular coagulation, and acute kidney injury. Plasma exchange therapy and continuous hemodiafiltration are likely to be effective treatments for ABO-incompatible blood transfusion, and further studies are required to assess this effectiveness in future.

## Introduction

ABO-incompatible blood transfusion often causes acute hemolytic reaction followed by disseminated intravascular coagulation (DIC) and acute kidney injury (AKI). In a report by Kim et al., symptoms occurred in 64% of the patients who were transfused with ≥ 50 mL ABO-incompatible blood, and the mortality rate was 17% [1]. The current treatment consists of prompt discontinuation of the blood transfusion along with anti-DIC treatments, in cases of DIC. However, there are few reports on plasma exchange therapy for ABO-incompatible blood transfusion. We report a case of 74-year-old male, who was accidentally transfused with ABO-incompatible blood, treated by plasma exchange therapy and continuous hemodiafiltration (CHDF).

## Case presentation

A 74-year-old man was admitted to hospital for the treatment of ascending colon cancer. Previous lower gastrointestinal endoscopy revealed 5-cm lesion in the ascending colon, and the biopsy results confirmed colorectal adenocarcinoma. The staging of tumor was cT1N0M0, making it an ideal candidate for treatment with laparoscopy-assisted partial excision of the ascending colon.

Considering the patient’s past medical history, he underwent distal gastrectomy for gastric cancer at 45 years of age and prostatectomy for prostate cancer at 70 years of age. He suffered from chronic hepatitis secondary to hepatitis C. On admission, his medication included candesartan 8 mg and amlodipine 5 mg for hypertension, and allopurinol 100 mg for hyperuricemia. His body weight was 63.7 kg. Blood test findings prior to admission showed microcytic hypochromic anemia with an Hb of 8.5 g/dL with normal platelet count and clotting time (Table 1).

**Table 1:** Laboratory findings on admission

Urinalysis		Full blood count			Biochemistry	
Specific gravity	1.024	WBC		5100 /µL	Na	146 mmol/L
pH	5.5	RBC		310 × 10^4^/µL	K	3.8 mmol/L
Protein	–	Hb		8.5 g/dL	Cl	109 mmol/L
Blood	–	Hct		27.6%	BUN	14.8 mg/dL
Bilirubin	–	MCV		89.0 Fl	Cre	0.85 mg/dL
		MCH		27.4 Pg	TP	6.2 g/dL
		MCHC		30.8%	ALB	3.8 g/dL
		Plt		17.9 × 10^4^/µL	UA	5.3 mg/dL
		Coagulation			T-Bil	0.5 mg/dL
		PT		84%	LDH	132 IU/L
		PT-INR		1.08	AST	18 IU/L
		APTT		25.6 s	ALT	11 IU/L
					ALP	237 IU/L
					γGTP	25 IU/L
					CK	88 IU/L
					CRP	< 0.3 mg/dL

The laparoscopy-assisted partial excision of the ascending colon was eventually converted to laparotomy due to multiple adhesions at 90 min into the operation. Blood transfusion was performed at 180 min into the operation because of increased hemorrhage. The patient’s blood type was O Rh positive; however, he was accidentally transfused with 280 mL (2 units) of type B Rh-positive blood.

At 17 min after the commencement of the ABO-incompatible blood transfusion, the patient’s blood pressure decreased from 150/70 to 90/40 mmHg. There was increased bleeding in the surgical area, and the amount of bleeding increased. Catecholamines were administered, and 1400 mL (10 units) of type O blood and 1200 mL (10 units) of type AB fresh frozen plasma (FFP) were transfused. At approximately 120 min after the incompatible blood transfusion, hemoglobinuria was detected.

The surgery was eventually completed, and the patient was transferred to the intensive care unit (ICU) approximately 200 min after the incompatible blood transfusion. Catecholamine treatment was continued to maintain hemodynamics, and an artificial respiratory treatment was instituted.

Hemoglobinuria suggested a high possibility of acute hemolytic reaction, DIC, and AKI due to the incompatible blood transfusion. Plasma exchange therapy and CHDF were initiated to improve the above-mentioned conditions as soon as the patient entered the ICU. The plasma separator used for plasma exchange therapy was OP-05W (Asahi KASEI). During plasma exchange therapy, 1–1.5 times of the actual plasma volume is generally exchanged. In our case, the patient’s body weight was 63.7 kg, his hematocrit was 27.6%, and his plasma volume was 3.23 L on calculation. However, we used larger amount of FFP because this case was severe. We exchanged 13.84 L FFP and performed plasma exchange therapy for 12 h while observing the improvement in plasma color and decrease in free hemoglobin levels. On CHDF, the hemofilter used was PUT-09eco (NIPRO), blood flow rate (*Q*_b_) was 80 mL/min, dialysis flow rate (*Q*_d_) was 400 mL/min, and filtration flow rate (*Q*_f_) was 300 mL/min. SUBPACK-Bi (NIPRO) was used for diafiltration and nafamostat mesilate was used as an anti-coagulant. We used UT-1100S (NIPRO) as the hemofilter because of coagulation of the previous hemofilter. We performed CHDF for 23 h. In addition, 7.56 L type O red blood cells, 2.55 L type O platelets, and 12.2 L type AB FFP were transfused repeatedly for up to 72 h. Steroid was used to inhibit acute hemolytic reaction, and 2000 mg/day gabexate mesilate, a protease inhibitor, was administered as anti-DIC therapy.

**Fig. 1:**
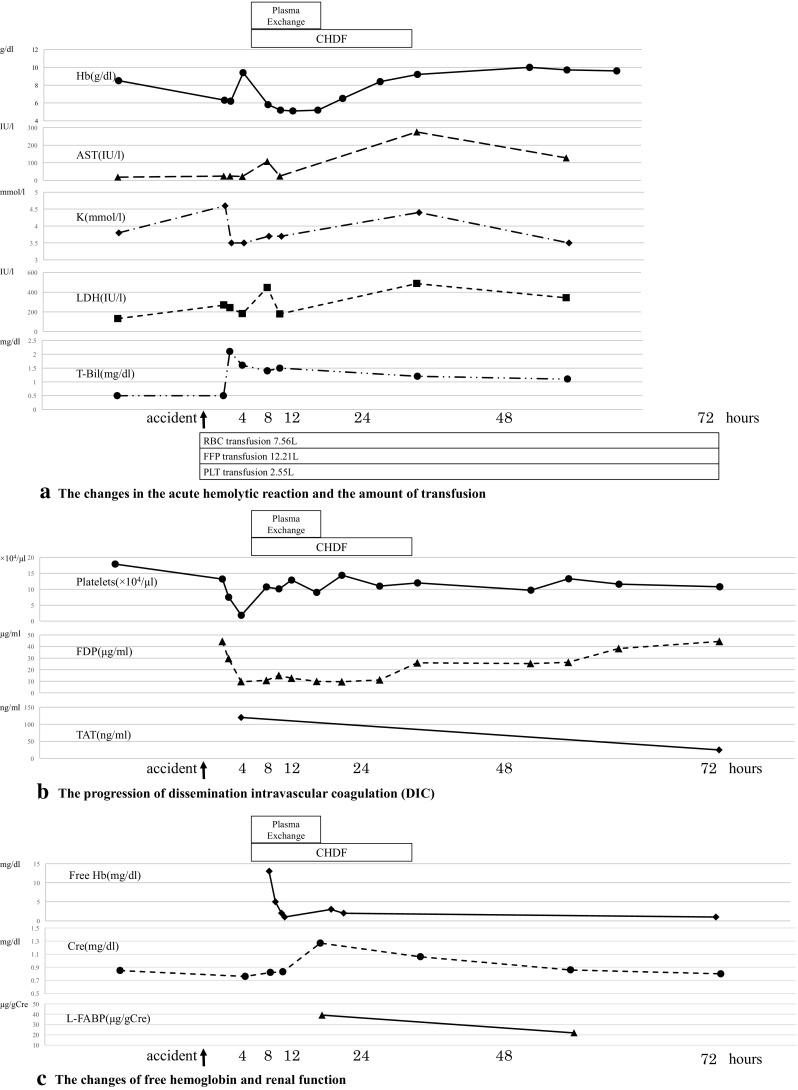
The overview of the incompatible blood transfusion

At 420 min after the incompatible blood transfusion, anemia progressed with a decline in Hb to 5.8 g/dL. In addition, increased lactic acid dehydrogenase (LDH), total bilirubin (T-Bil), and aspartate aminotransferase (AST) suggested the presence of hemolytic anemia (Fig. 1a). The platelet count reached 1.8 × 10^4^/µL, fibrin/fibrinogen degradation product (FDP) concentration was 9.7 µg/mL, thrombin–antithrombin complex (TAT) was 120 ng/mL, and plasmin–α2 plasmin inhibitor (PIC) complex was 0.4 µg/mL (Fig. 1b). The blood results were consistent with the characteristics of DIC. DIC due to acute hemolytic reaction was responsible for the bleeding tendency, which worsened further post-surgery. The amount of bleeding was 9.13 L up to 72 h.

At 48–72 h after the accident, T-Bil and LDH levels decreased and Hb increased to approximately at 9.0 g/dL, suggesting improvement in hemolytic anemia. The serum haptoglobin was 26 mg/dL at 48 h. DIC improved with decreasing TAT when the platelet count was approximately 11 × 10^4^/µL after plasma exchange therapy and CHDF (Fig. 1b).

**Fig. 2:**
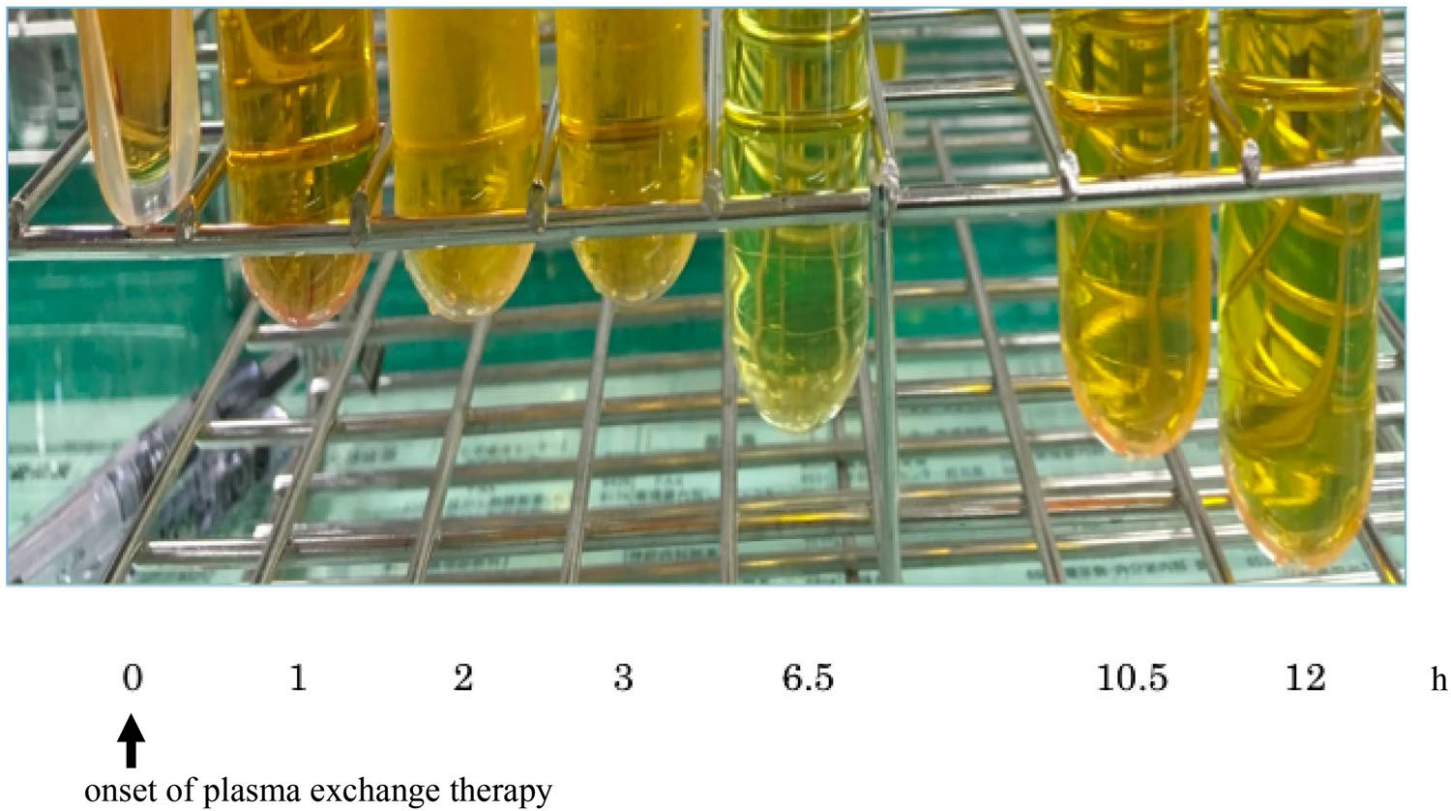
Changes in the color of plasma

**Fig. 3:**
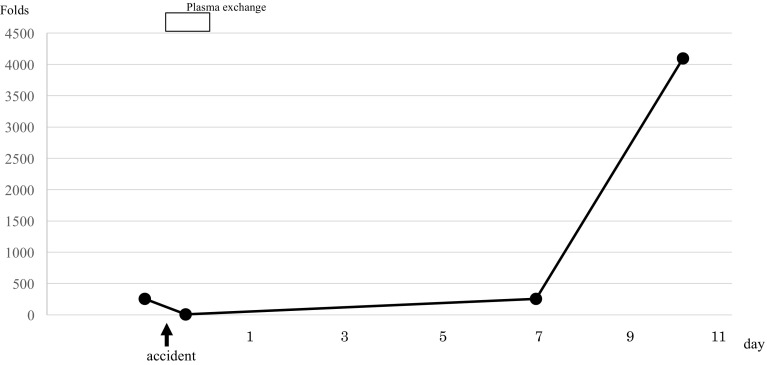
Changes in anti-B antibody levels

Free hemoglobin and anti-B antibody levels were measured over time. After the initiation of the plasma exchange therapy, the level of free hemoglobin decreased rapidly with the color of plasma changing from cloudy yellow to clear yellow (Figs. 1c, 2). Although anti-B antibody levels increased by 256-fold immediately after the accident, this decreased transiently as the antibody was consumed by the hemolytic reaction. It remained at a low value thereafter, and did not show an increase to its level even on day 7 post-blood transfusion (Fig. 3).

From day 6 after the incompatible blood transfusion, the hemolytic reaction worsened, with increasing T-Bil and LDH levels and a decreasing haptoglobin level. We started to infuse haptoglobin 4000 units/day when the serum haptoglobin was 6 mg/dL at day 9. The anti-B antibody levels also increased. The hematoma containing residual B antigens around the liver was suspected to contribute to the worsening of the hemolytic reaction. The hematoma was, thus, surgically drained on day 18. Levels of T-Bil, LDH, haptoglobin, and FDP subsequently improved.

The patient maintained a good urinary output and his initial serum creatinine was 0.76 mg/dL at the beginning of CHDF. Subsequently, the level of the urinary liver-type fatty acid-binding protein (L-FABP) increased to 39.1 µg/gCre, and the serum creatinine was increased to 1.25 mg/dL with newly onset proteinuria (Fig. 1c). The results suggested AKI. Plasma exchange therapy and CHDF were commenced with subsequent rapid improvement of brown discoloration of urine, and rapid normalization of serum creatinine and L-FABP levels (Fig. 1c).

Ventricular fibrillation occurred on day 24 and treatment for infection due to carbapenem-resistant enterobacteriaceae (CRE) and multiple-drug-resistant *Pseudomonas aeruginosa* (MDRP) was required for 60 days. Incompatible blood transfusion was completely cured on day 58 because FDP was normal on that day. The patient underwent a long period of rehabilitation upon request of the patient’s family. As this was a case of a medical accident, we took considerable care of the patient. The patient was discharged on day 151 after the incompatible blood transfusion.

## Discussion

The rate of mortality from ABO-incompatible blood transfusion reported by Kim et al. [1] and Linden et al. [2] was 14 and 5.5%, respectively. Kim et al. further reported that when ≥ 50 mL blood was transfused, symptoms occurred in 64% of the patients, and death occurred in 17%. On the contrary, when less than 50 mL blood was transfused, symptoms occurred in only 25% of the patients with no reported deaths [1].

When type B RBCs are transfused to a type O RBC patient, the patient’s own anti-B antibodies react with the B-antigen on the type B RBC surface. This antigen–antibody reaction leads to complement reaction, which causes systemic inflammatory reaction such as fever, hypotension and DIC, and hemolysis, which results in the release of free Hb causing DIC and AKI [1, 3].

Complement activation triggered by the antigen–antibody reaction has a direct effect not only on the hemolysis but also on the systemic inflammatory reaction. In particular, C3a and C5a bind to the C3a and C5a receptors that are expressed on lymphocytes, macrophages, and vascular endothelial cells. This reaction is followed by the production of free radicals, nitric oxide (NO), leukotrienes, and interleukins [4, 5]. When this occurs in excess, they trigger systemic inflammatory reactions, such as hypotension due to increasing vascular permeability [6] and fever. On mixing of type A or B blood cells with type O whole blood, IL-8 and TNF levels were reported to increase in in vitro experiments [7, 8]. TNF promotes the release of tissue factors from vascular endothelial cells, resulting in further decrease in thrombomodulin responsible for DIC [9]. Thus, complement activation is also responsible for DIC.

Free hemoglobin enhances vascular permeability, triggering the activation of white blood cells [6]. This leads to further induction of clotting cascade and systemic inflammatory reactions [3]. Free hemoglobin also binds and inhibits NO, resulting in constriction of the vascular smooth muscles, platelet activation, and platelet clumping [10, 11]. These mechanisms are consequently responsible for DIC.

AKI is caused by DIC and renal tubular impairment owing to the presence of free hemoglobin [3, 12, 13]. In an experimental monkey model, fibrin clotting was observed within the kidney after an incompatible blood transfusion that suggested the involvement of DIC induced by acute hemolytic reaction [14]. Hemoglobinuria was recently reported to impair the renal tubular barrier, causing oxidative damage to renal tubular cells [12] and the reduction in density of the peritubular capillaries (PTC) [13].

The treatment for incompatible blood transfusion involves a prompt discontinuation of blood transfusion and administration of appropriate supportive therapy. No other clear treatment modality has been established. In recent reports, whole blood exchange transfusion [15] and use of heparin and eculizumab [16] were described; however, there is no established evidence of these treatments. As far as we are aware, no studies on plasma exchange therapy have been reported to date (Table 2).

**Table 2:** Reports on incompatible blood transfusion

Patient	Blood type	Type of transfused blood and amount	Complication	Treatment	Prognosis	References
32-year-old male	O	Type A, 450 mL	Hypotension, acute hemolysis	None	Survived	Janatpour et al. [1]
36-year-old female	O	Type A, 300 mL	Hypotension	Support therapy only	Survived	Janatpour et al. [1]
53-year-old female	O	Type A, 600 mL	Fever, increased blood pressure, acute hemolysis, acute renal impairment	Support therapy only	Died	Janatpour et al. [1]
60-year-old male	O	Type A, 1200 mL	Hypotension, acute hemolysis, acute renal impairment, DIC	Support therapy only	Died	Janatpour et al. [1]
65-year-old male	O	Type A, 600 mL	Acute hemolysis, acute renal impairment	Support therapy (hemodialysis for 2.5 weeks)	Survived	Janatpour et al. [1]
78-year-old male	O	Type B, 300 mL	None	None	Survived	Janatpour et al. [1]
80-year-old male	O	Unknown	Hypotension, acute hemolysis	Support therapy only	Died	Janatpour et al. [1]
81-year-old male	A	Type B, 300 mL	Unknown	Support therapy only	Died	Janatpour et al. [1]
PNH patient	B	Type A, 300 mL	Acute hemolysis only	Eculizumab, heparin	Survived	Weinstock et al. [16]
64-year-old female	A (anti-e antibody present)	Anti-e antigen-positive type A, 9600 mL	Hypotension, acute hemolysis	Exchange blood transfusion	Survived	Irani et al. [15]

1 unit = 300 mL

Plasma exchange therapy involves the replacement of plasma in the blood with FFP or 5% albumin to remove toxic substances in the plasma and supplement insufficient substances [17]. For incompatible blood transfusions, plasma exchange therapy results in the removal of anti-A or anti-B antibodies and the removal of free hemoglobin.

The removal of anti-A or anti-B antibodies can inhibit the antigen–antibody reaction. In ABO-incompatible renal transplants, anti-A or anti-B antibodies are removed by plasma exchange therapy, resulting in successful transplantation. The study by Clark et al. revealed that the lower antibody titer is associated with improved grafting rate [17]. In our case, anti-B antibody titer was low during plasma exchange therapy. We detected type B RBCs from incompatible transfusion at the end of plasma exchange therapy. This suggested that there had been lasting hemolytic reaction during plasma exchange therapy and there had been undestroyed type B RBCs at the end of plasma exchange therapy. As AST, LDH, and T.Bil levels were not elevated during plasma exchange therapy, removal of anti-A or anti-B antibodies may help to minimize antigen–antibody reaction and hemolysis in incompatible blood transfusions.

Removal of free Hb can inhibit DIC and AKI. Duvall et al. described a case where 140 mL of type A Rh-positive blood was accidentally transfused to a type O patient and the time required to reach the minimum free hemoglobin level was approximately 24 h [18]. In our case, free hemoglobin levels decreased from 13 to 2 mg/dL for 2 h (Fig. 1c). We demonstrated that a rapid decrease in free hemoglobin could be achieved within 2 h using plasma exchange therapy and thus gained improvement of DIC and AKI.

CHDF may be a useful adjunct to stabilize hemodynamics during incompatible blood transfusion, as it helped to avoid volume overload. CHDF enabled the patient to be transfused 7.56 L RBCs for 72 h for treatment. This large blood transfusion amount was similar to the whole blood exchange transfusion, which may thus be effective in incompatible blood transfusion.

In conclusion, we described a case of incompatible blood transfusion successfully treated with plasma exchange therapy and CHDF. This report shows that plasma exchange therapy and CHDF are potentially useful for inhibiting the onset and progression of acute hemolytic reactions, DIC, and AKI.

## Abbreviations

AKI: Acute kidney injury
AST: Aspartate aminotransferase
CRE: Carbapenem-resistant enterobacteriaceae
CHDF: Continuous hemodiafiltration
DIC: Disseminated intravascular coagulation syndrome
FDP: Fibrin/fibrinogen degradation product
FFP: Fresh frozen plasma
ICU: Intensive care unit
LDH: Lactic acid dehydrogenase
L-FABP: Liver-type fatty acid-binding protein
MDRP: Multiple-drug-resistant *Pseudomonas aeruginosa*
NO: Nitric oxide
PTC: Peritubular capillaries
PIC: Plasmin-α2 plasmin inhibitor
TAT: Thrombin–antithrombin complex
T-Bil: Total bilirubin
